# VDD Lead Extraction—Differences with Other Leads and Practical Tips in Management

**DOI:** 10.3390/jcm13030800

**Published:** 2024-01-30

**Authors:** Andrzej Kutarski, Wojciech Jacheć, Paweł Stefańczyk, Anna Polewczyk, Jarosław Kosior, Dorota Nowosielecka

**Affiliations:** 1 Department of Cardiology, Medical University of Lublin, 20-059 Lublin, Poland; a_kutarski@yahoo.com; 2 2nd Department of Cardiology, Faculty of Medical Sciences in Zabrze, Medical University of Silesia, 40-055 Katowice, Poland; 3 Department of Cardiology, The Pope John Paul II Province Hospital of Zamosc, 22-400 Zamosc, Poland; 4 Department of Medicine and Health Sciences, The John Kochanowski University, 25-369 Kielce, Poland; 5 Department of Cardiac Surgery, Świętokrzyskie Center of Cardiology, 25-736 Kielce, Poland; 6 Department of Cardiology, Masovian Specialistic Hospital of Radom, 26-617 Radom, Poland; 7 Department of Cardiac Surgery, The Pope John Paul II Province Hospital of Zamosc, 22-400 Zamosc, Poland

**Keywords:** VDD lead extraction, transvenous lead extraction, difficult lead extraction, complexity of lead extraction

## Abstract

**Background:** VDD (atrial sensing, ventricular sensing/pacing) leads are relatively rarely implanted; therefore, experience in their extraction is very limited. We aimed to investigate whether VDD lead removal may be a risk factor for the increased complexity of transvenous lead extraction (TLE) or major complications. **Methods:** We retrospectively analyzed 3808 TLE procedures (including 103 patients with VDD leads). **Results:** If TLE included VDD lead removal, procedure duration (lead dilation time) was prolonged, complicated extractions were slightly more common, and more advanced tools were required. This is partly due to longer implant duration (in patients with VDD systems—135.2 months; systems without VDD leads—109.3 months; *p* < 0.001), more frequent presence of abandoned leads (all systems containing VDD leads—22.33% and all systems without VDD leads—10.77%), and partly to the younger age of patients with VDD leads (51.74 vs. 57.72 years; *p* < 0.001, in the remaining patients) at the time of system implantation. VDD lead extraction does not increase the risk of major complications (1.94 vs. 2.34%; *p* = 0.905). **Conclusions:** The extraction of VDD leads may be considered a risk factor for increased procedure complexity, but not for major complications. However, this is not a direct result of VDD lead extraction but specific characteristics of the patients with VDD leads. Operator skill and team experience combined with special custom maneuvers can enable favorable results to be achieved despite the specific design of VDD leads, even with older VDD lead models.

## 1. Introduction

Permanent VDD (atrial potential-controlled ventricular inhibited) pacing using a single ventricular lead with two floating rings at the atrial level for the detection of atrial potentials was introduced more than 30 years ago [1,2,3,4,5]. This kind of pacing system is designed for patients with atrioventricular block and normal sinus node function [6]. It allows the maintenance of AV synchronous pacing and its hemodynamic advantages [3,4,7]. The disadvantages include a relatively frequent atrial undersensing in 4% to 16% [8,9,10,11] and a risk of sinus node dysfunction with a subsequent need for an upgrade to DDD pacing [8,12]. In spite of the simplicity of implantation, shorter fluoroscopy time, and lower complication rate compared to the implantation of DDD pacemakers [12,13], the use of this type of pacing mode is currently low in daily clinical practice; e.g., in a recent Italian national registry, only 5.9% of pacemakers implanted for AVB were VDD devices [14].

Transvenous lead extraction (TLE) plays a key role in the management of lead/system-related problems (infections, lead replacements, or system upgrades). It is a highly effective (over 95%) procedure, though it is associated with major complications in 1.6–2.5% [15,16]. Its goal is to remove the leads integrally with minimal risk of major complications (influenced mainly by operator experience and tools) and complication-related deaths (proper organization of the procedure should reduce the risk of its occurrence) [15,16]. But the procedure once started must be completed even if difficulties are encountered (e.g., break of the targeted lead). Prediction and management of major complications have been the subject of numerous reports [17,18,19,20]. Increased procedure difficulty was defined as prolonged procedure or fluoroscopy time [18,19,20,21,22,23,24,25,26], the need for advanced tools and methods [24,25,26,27,28], or an increased number of laser pulses delivered [20,23].

The type of lead to be removed (ICD, passive fixation, silicone) was considered one of the risk factors for the increased complexity of the procedure and the occurrence of major complications [27,28,29,30,31].

VDD systems have limited applications [6,8,12,13]. Therefore, they are rarely implanted [14], and in studies on lead extraction, VDD leads are taken together with other PM leads. In the literature on TLE, we have found only three papers in which the proportion of VDD leads was provided, ranging from 3.5 to 4.5% [32,33].

VDD leads have four conductors and three ring electrodes and, therefore, have a more delicate structure than standard PM leads [1,2,3,4,5]. Two atrial electrodes with rings, often in constant contact with the atrial wall [1,2,3,4,5], suggest that such electrodes may be more difficult to remove.

We found only one report in the literature demonstrating a relationship between the lack of procedural success and the presence of VDD leads [34]. However, the investigators made their observations after analyzing only eleven VDD leads. Other aspects of VDD lead extraction, apart from the lack of procedural success due to technical problems, procedure complexity, and major complications, were not analyzed [34].

However, the investigators made their observations after analyzing eleven VDD leads. Other aspects of VDD lead extraction were not analyzed [34]. Also, based on the results of our previous research [33], we have concluded that the issue of VDD lead extraction deserves a broader study.

Having a database of 3808 TLEs enabled an in-depth analysis of the transvenous extraction of relatively rarely implanted and rarely removed VDD leads.

### What Is New?

Transvenous lead removal should be completed successfully and without complication-related deaths. Awareness of unexpected “pitfalls” that increase the difficulty of the procedure may be insufficient. The type of electrode being removed may play an important role. VDD electrodes are relatively rarely implanted and removed, so understanding the specifics of their extraction procedure is limited. This is the first paper describing the difficulties and specificity of VDD electrode extraction, which is more troublesome and laborious (lead dilatation duration) than normal but does not carry an increased risk of major complications. The complexity of TLE in patients with VDD systems or VDD abandoned leads is related to the older age of the implanted leads and their passive fixation. Two floating (only theoretically) annular atrial electrodes are often in constant contact with the atrial wall, and scar tissue at this site is sometimes harder and more difficult to pass through. The car tissue on atrial floating annular electrodes makes lead dilation difficult. This is the usual site of conductor externalization and secondary lead damage during its dissection from the scar. A change of the dilator sheet for a larger one is often necessary. The pulling on the lead during its dilatation slightly reduces the diameter of the lead, which makes it possible for the ring electrodes to move until they fall completely off the lead. VDD lead extraction requires the highest quality fluoroscopy, operator, and team attention. The specificity of the extraction of such electrodes should be known to operators.

## 2. Goals of This Study

The aim of this study was to provide a comprehensive description of VDD lead extraction and find out how often the leads of VDD systems are removed, remain functional in upgraded systems, or are abandoned. We decided to compare the course and outcomes of lead removal between patients with VDD leads and patients with VVI, DDD, and other systems (ICD, CRT-D, and AAI) in terms of patient-dependent, system-dependent, and procedure-dependent risk factors for a difficult and complicated procedure. Overall, the main objective was to investigate whether the presence of a VDD lead is a risk factor for increased procedure complexity or major complications.

## 3. Methods

### 3.1. Study Population

All transvenous lead extraction (TLE) procedures performed between March 2006 and December 2022 at a single high-volume center were reviewed. Patient clinical data, information on CIEDs and history of pacing, extracted leads, extraction complexity, efficacy, and outcomes were retrospectively analyzed from our computerized database. This study population included 3808 patients (38.1% females), aged 5–97 years, with an average age of 66.0 years.

### 3.2. Lead Extraction Procedure

Indications for TLE, procedure effectiveness, and complications were defined according to the recent recommendations (2009 and 2017 HRS consensus and 2018 EHRA guidelines) [15,16]. The efficacy of TLE was expressed as the percentage of procedural success, which was defined as the removal of all targeted leads and lead material from the vascular space in the absence of any permanently disabling complication or procedure-related death [15,16].

The complications of TLE were also defined as major complications, being those that were life-threatening, resulted in significant or permanent disability or death, or required surgical intervention [15,16].

The risk of major complications (MC) related to TLE (points, percentage) was assessed using the SAFeTY TLE score, an online tool available at (https://usuwanieelektrod.pl/akalkulator/) accessed 5 December 2023 [18]. The EROS score was used for the prediction of significant procedural complications that required emergent surgical intervention (1–3 points) [27]. Assessment of procedure complexity was based on the MB score showing the need for the use of advanced tools to achieve TLE success (0–5 points) [28], the LED index referring to lead extraction difficulty based on fluoroscopy times (0–50 points) [29], and the Advanced TLE Techniques (Mazzone) score to predict the necessity of using advanced extraction techniques (0–4 points) [30].

Procedure complexity was expressed as lead extraction time (sheath-to-sheath time) and the average time of single lead extraction (sheath-to-sheath/number of extracted leads). The second indicator of procedure complexity was the necessity of using second-line tools and advanced tools [19,20,21,22,23,24,25,26,31]. The third complexity marker was The Complex Indicator of the Difficulty of the TLE (CID-TLE), which includes global sheath-to-sheath time (extraction of all leads) >20 min (2 points), average duration of single lead extraction (sheath-to-sheath time) >12 min (2 points), and the necessity of using metal sheaths or rotational mechanical dilators (Evolution^®^/TightRail^®^), alternative approaches, or lasso-catheters or basket catheters (for 1 point each). The sum of points was the value of CID-TLE. The procedure was deemed to be difficult when CID-TLE was ≥2 [31].

Unexpected procedure difficulty caused so-called technical problems during TLE, i.e., situations that increased procedure complexity but were not complications [35]. They included break of extracted lead [26,35,36,37,38,39,40,41,42,43,44], loss of broken lead fragments when the main part of the lead was dilated and removed but both free ends remained in place, mobile lead fragments that flowed usually into the pulmonary vascular bed [28,35,39], blockage in lead venous entry/subclavian region preventing entry into the subclavian vein with a polypropylene catheter, Byrd dilator collapse/fracture [35], lead-to-lead adhesion [35,40], necessity of using an alternative approach [35,40] and displacement of functional leads [35,40].

### 3.3. Dataset and Statistical Methods

#### 3.3.1. Creation of the Subgroups for Analysis of Events and Patients

The entire group of 3808 TLE procedures was divided into five subgroups: 1. TLE in patients with VDD leads (active or abandoned)—103 procedures; 2. TLE in patients with AAI PM systems—273 procedures; 3. TLE in patients with VVI pacing systems (with or without abandoned leads but without VDD leads)—470 procedures 4. TLE in patients with DDD or CRT-P PM systems (with or without abandoned leads but without VDD leads)—1866 procedures and 5. TLE in patients with defibrillation lead/s (ICD-V, ICD-D, CRT-D with or without abandoned leads but without VDD leads)—1096 procedures (Table 1). We are aware of the fact that the presence of abandoned leads in all groups affects the final result; however, excluding patients with abandoned leads from further analysis would reduce the material by 1/3 and show a non-representative, non-existing, trivial practice group of patients. Excluding patients with abandoned leads from further analysis would reduce the material by 1/3 and show a non-existent “ideal world”. Most often, the follow-up data were obtained by telephone contact. In cases of lack of contact with the patient or his family, it was data from the government population register.

#### 3.3.2. Statistical Methods

For uniformity, all continuous variables are presented as the mean ± standard deviation. The categorical variables are presented as numbers and percentages. The significance of differences between the VDD group and groups without VDD lead was determined using the non-parametric Chi^2^ test with Yates correction or the unpaired Mann–Whitney U test, as appropriate. For comparison of the groups for statistical significance evaluated with the Mann–Whitney U and Chi^2^ tests, the Bonferroni correction was applied, considering the value of *p* < 0.0125 as statistically significant. To determine the impact of extracted leads on procedure complexity, clinical success, and the presence of major complications, univariable and multivariable regression analyses were used. The variables achieving *p* < 0.05 under a univariable regression model were entered into a multivariable model. A *p*-value less than 0.05 was considered statistically significant. Statistical analysis was performed with Statistica 13.3 (TIBCO Software Inc., Palo Alto, CA, USA).

#### 3.3.3. Approval of the Bioethics Committee

All patients gave their informed written consent to undergo TLE and use anonymous data from their medical records, approved by the Bioethics Committee at the Regional Chamber of Physicians in Lublin, no. 288/2018/KB/VII (the date of issue: 27 November 2018). This study was carried out in accordance with the ethical standards of the 1964 Declaration of Helsinki.

## 4. Results

Among 103 patients with removed VDD leads, the lead continued to function as a VDD lead in 72 (69.9%), as a VVI lead in DDD systems in 13 (12.62%), as a VVI lead in CRT-P systems in 3 (2.91%), and as an abandoned lead in the remaining 15 (14.46%). The mean implant duration was longer in non-VDD systems (143.29 months) than in still-functioning VDD systems (129.89 months) and in patients without VDD lead removal (99.55 months). In patients with still-functioning VDD systems, the main indication for TLE was mechanical lead damage or lead dysfunction for other reasons (55.55%), and in patients with a VDD lead but with a different type of pacing system, this percentage was lower (37.71%) because frequently there were other non-infectious indications (25.81%). Noteworthy is the higher percentage of abandoned leads (22.33%) in patients with VDD leads than in patients without VDD leads (10.77%) (Table 1).

Analysis of patient clinical data showed that age during TLE and at first system implantation were lower in patients with removed VDD leads compared to patients with AAI or DDD/CRTP systems. The mean LVEF in the VDD group was lower compared to the AAI group and higher compared to the ICD/CRTD group. In contrast, the NYHA class and Charlson co-morbidity index in the VDD group were lower than in the ICD/CRTD group. There were no significant differences in infectious indications for TLE between the VDD group and other groups.

Summing up, patients with removed VDD pacing leads were younger at the time of their first CIED implantation and had fewer co-morbidities compared to patients with other leads (Table 2).

Patients with systems other than VDD, AAI, and VVI had younger leads, fewer abandoned leads, fewer leads located on both sides of the chest, and fewer CIED-related procedures.

We made an attempt to predict procedure difficulty using TLE complexity risk scales/calculators. While the EROS and SAFeTY TLE scales dedicated to prediction of major complication risk did not significantly differ between the groups (except the ICD-CRTD group), the calculators for prediction of increased procedure complexity showed an increased level of procedure complexity if the removal of VDD leads was planned. Calculators such as MB score [need for advanced tools], LED index [predicted fluoroscopy time], Advanced TLE (ALE) scale [need for advanced TLE techniques], and LECOM score (increased procedure complexity) showed significant differences between the VDD lead removal group and patients with AAI (LECOM), VVI (MB, LED, ALE, LECOM), DDD or CRTP (LED, ALE, LECOM), and ICD-CRTD (LED, ALE, LECOM) systems without VDD leads. The greatest differences were found in the LECOM score.

The bottom part of Table 3 shows that extraction-related potential risk factors for major complications and procedure complexity, such as extraction of abandoned leads (except patients with VVI systems), extraction of passive fixation leads, and long dwell time of extracted leads (except patients with AAI or VVI systems), were significantly higher in the group of patients with removed VDD leads.

Analysis of TLE complexity showed that extraction procedures were often more difficult in the group with removed VDD leads, especially compared to procedures in patients with ICD-CRTD systems (Table 4).

Table 5 summarizes the incidence of major complications (any), haemopericardium, haemothorax, tricuspid valve damage during TLE, and the need for rescue cardiac surgery in this study groups. Analysis did not confirm that VDD lead extraction was associated with a higher risk of major complications. The extraction of VDD leads was not related to the occurrence of procedure-related deaths. Partial radiographic success (retained tip or <4 cm lead fragment) or chances of achieving procedural success were higher in the ICD-CRTD group compared to the VDD and other groups. Most deaths during follow-up occurred in the group of patients with removed VVI and ICD-CRTD systems (the oldest patients with the highest rate of multimorbidity).

Table 6 shows that there is no direct relationship between the complexity of the extraction procedure and the type of extracted lead, but there is a relationship between the presence of abandoned leads, the number of extracted leads, passive fixation leads, the younger age of patients at first CIED implantation, and the sum of lead dwell times. The achievement of procedural success was related to the number of leads, type of lead fixation, implant duration, patient age at first CIED, and TLE performed in patients with ICD-CRTD systems. In our model, only the younger age of patients at first CIED implantation and the older age of extracted leads were predictors of major complications.

### Author Comments on VDD Lead Extraction

**Figure 1:**
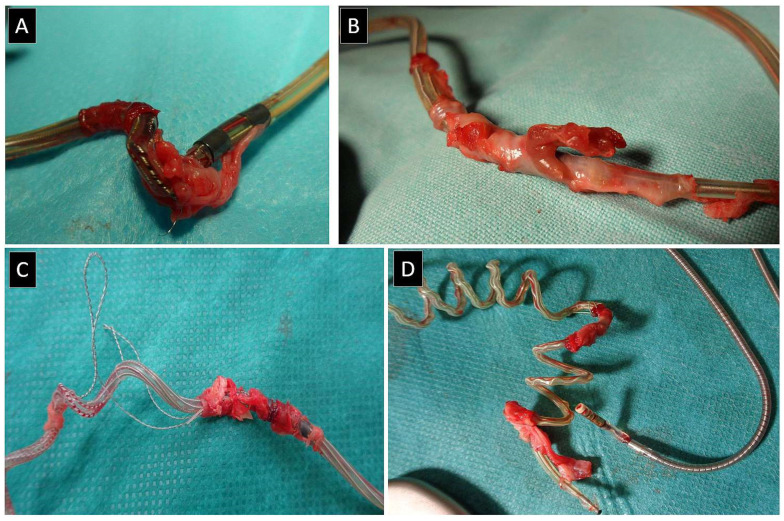
Different forms of scar tissue on the leads. Scar tissue and VDD leads (**A**–**D**). Different forms of scar tissue on atrial floating annular electrodes make lead dilation difficult. This is the usual site of conductor externalization and secondary lead damage during its dissection from the scar (**A**,**C**,**D**). The section of lead with floating rings often has permanent contact with the atrial wall, which results in the development of hardening connective tissue and the possibility of damage to the atrial wall during lead dilation. In the pictures, you can see the presence of muscle tissue, which is fragments of the atrial wall.

Current findings suggest that the extraction of pacemaker leads that includes the removal of VDD leads is only slightly more troublesome and laborious (lead dilatation duration) than normal but does not carry an increased risk of major complications. The complexity of TLE in patients with VDD systems or VDD abandoned leads is related to the older age of the implanted leads and their passive fixation. Two floating (only theoretically) annular atrial electrodes are often in constant contact with the atrial wall, and scar tissue at this site is sometimes harder and more difficult to pass through (Figure 1).

While this is undoubtedly true, the extensive experience of the first operator and the team must be taken into account. After uncovering the characteristics of VDD lead extraction, the efficiency of the procedures significantly improved. Peculiarities and practical aspects of VDD lead extraction should be discussed.

A change of the dilator sheath for a larger one is often necessary. Or, it is even better to start VDD lead extraction using a catheter one size larger than the standard PM lead. The pulling on the lead during its dilatation slightly reduces the diameter of the lead, which makes it possible for the ring electrodes to move until they fall completely off the lead.

**Figure 2:**
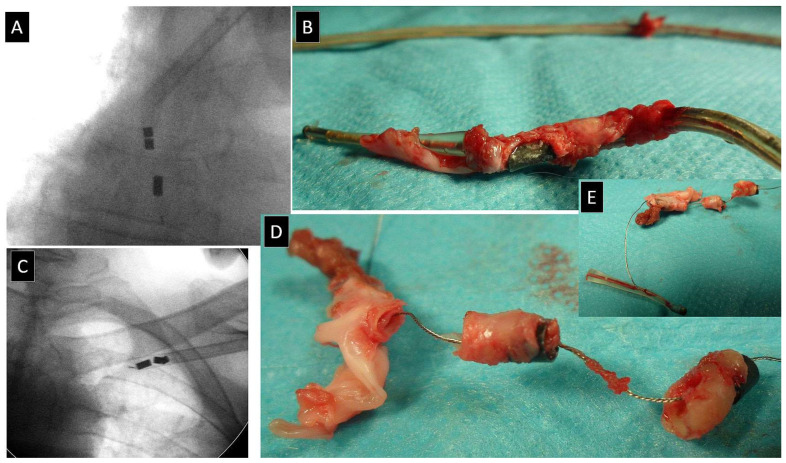
Extraction of typical old VDD leads. Sliding down distally to ring electrodes (atrial and anode ventricular electrodes) during lead dilatation—extraction of typical old VDD leads. Sometimes, they can be removed together with other leads (**A**–**D**), and sometimes, they are removed separately (**E**).

**Figure 3:**
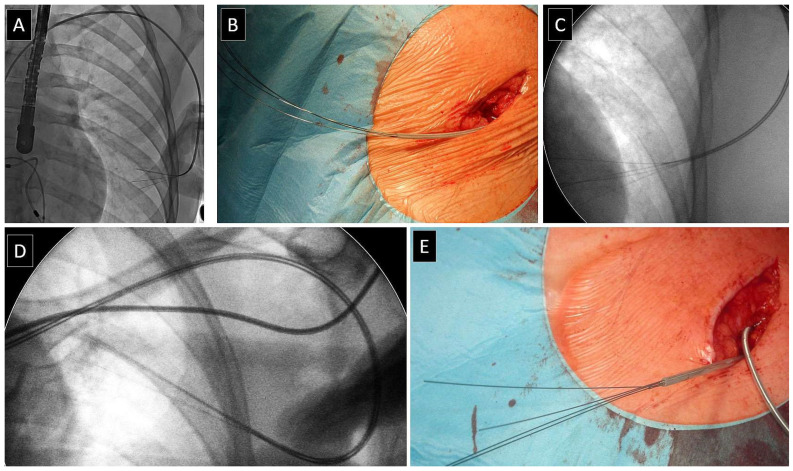
Construction of the older models of VDD and its impact on technique TLE. Older models of VDD leads have four independent helical conductors allowing the insertion of stylets (**A**–**E**). Stylet stiffen the lead by creating a splint (rail) for the dissecting catheter and reduce the risk of slipping off the ring electrodes. Sometimes the angulation of the leads makes it impossible to reach the end of the conductor with the stylet (**A**). The trick of introducing 4 styles is shown in 3 radiographs of 3 patients (**A**,**C**,**D**) and photographs of the surgical field (**B**,**E**).

VDD leads, especially floating-ring atrial electrodes, have a larger diameter than standard PM leads. Also, anode ventricular electrodes are longer than normal and have a tendency to slip off, similar to atrial annular electrodes (Figure 2). VDD lead extraction requires the highest quality fluoroscopy, operator, and team attention. Another feature of most (especially older) bipolar VDD leads is the construction of four conductors running parallel to each other in the form of a conventional spiral. It is, therefore, possible to stiffen the entire lead with four styles. The lead stiffened in this way is significantly less susceptible to sharp deflections and changes its thickness (diameter) to a lesser degree during necessary tensioning. This trick reduces the risk of slipping off the annular electrodes (two atrial and one ventricular) and the risk of collapse of the polypropylene catheter (Figure 3).

**Figure 4:**
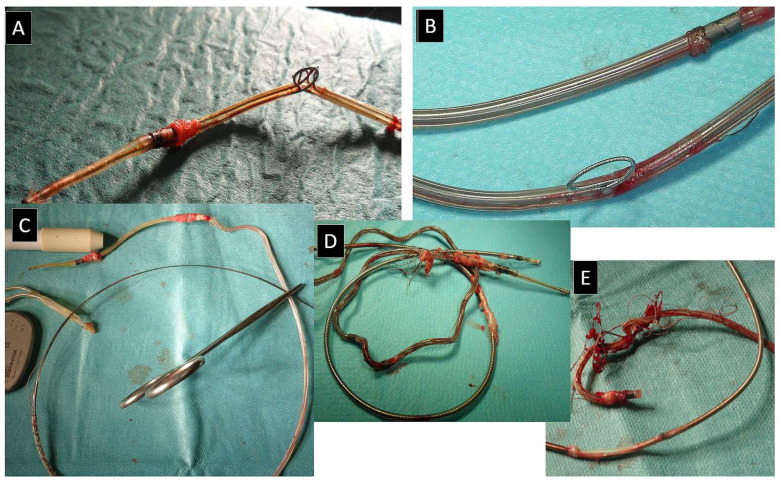
Examples of conductor externalization, which is relatively common phenomenon in VDD leads. Conductor externalization—a relatively common phenomenon in VDD—leads at the site of their abnormal deflection; which often occurs in the vicinity of atrial floating ring electrodes (**A**–**C**). Conductor externalization may contribute to lead dysfunction and hinder lead dilatation, and the removed leads can be significantly damaged in this area (**D**,**E**).

Difficult moments (technical problems) during VDD lead extraction occur slightly more often than during the extraction of standard leads, and they are often solved by replacing the catheter with another one having a larger diameter or changing the tool for a more effective one (mechanical rotational). An additional, though not so common, trap is the unrecognized externalization of conductors at the site of permanent deflection of the lead (Figure 4).

**Figure 5:**
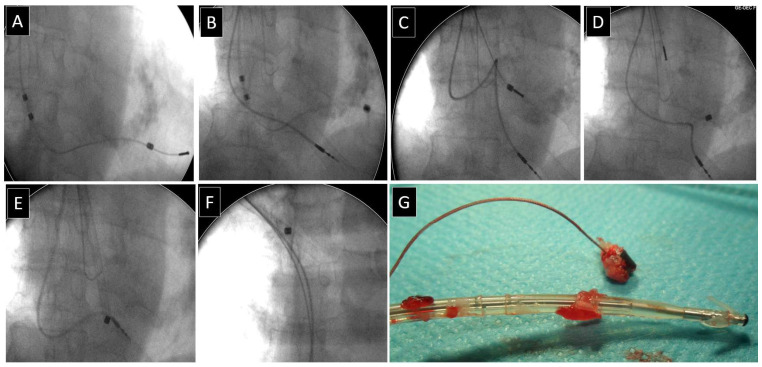
Slipping off the ventricular (ring) anode electrode during VDD lead removal. Slipping off the ventricular (ring) anode electrode during VDD lead removal (**A**–**D**). Since the electrode was connected to its conductor, it was possible to remove it at a later stage of the procedure (**E**–**G**).

**Figure 6:**
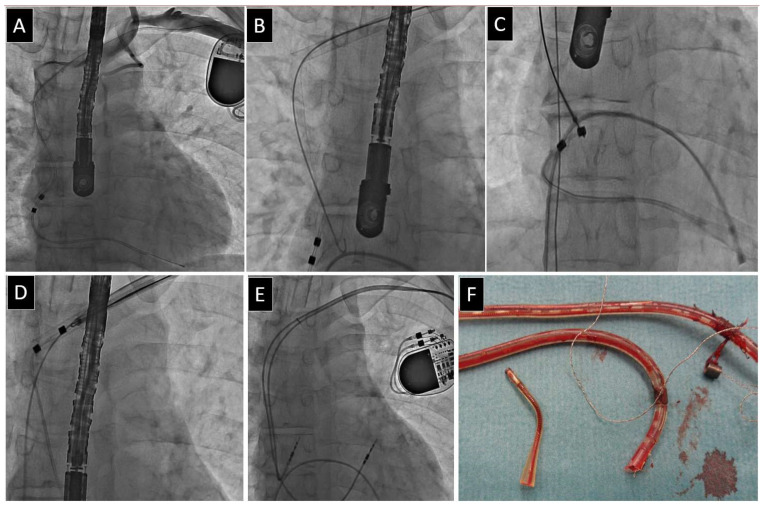
Example of rupture of VDD lead during extraction in patient with lead-related venous obstruction (**A**) complicated with polypropylene sheath collapse (**B**). The next complication was rupture of extracted VDD managed with a grab a lead fragment using accessed implant venous with a lasso catheter (**C**,**D**), which facilitated the effective removal of all fragments of the VDD lead (**D**,**F**) and the implantation of a new DDD system (**E**) despite venous obstruction (**A**).

**Figure 7:**
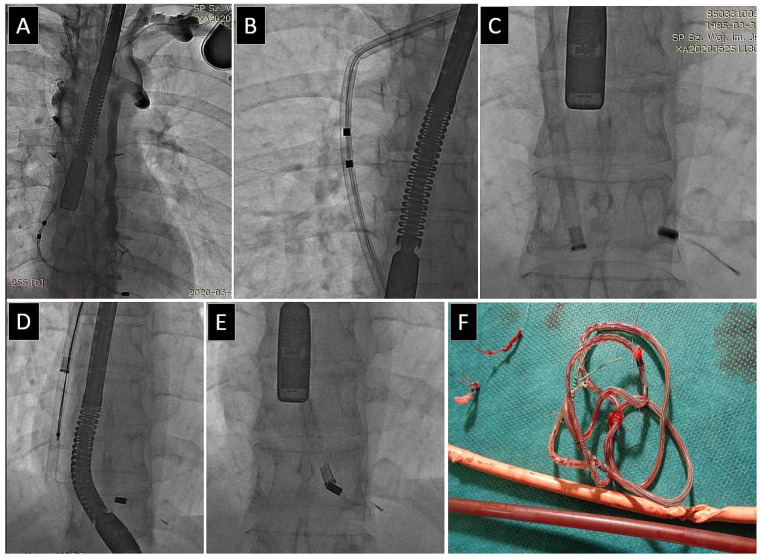
VDD lead extraction in a patient with lead-related venous obstruction. VDD lead extraction in a patient with lead-related venous obstruction (**A**). Polypropylene sheath collapse (**B**), repeat rupture of the VDD lead (**C**,**D**), and finally, incomplete removal (**E**,**F**).

In the event of VDD lead rupture, the procedure is identical to that in standard pacemaker lead rupture (Figure 5, Figure 6 and Figure 7).

## 5. Discussion

This study shows that if TLE included the removal of VDD leads, procedure duration (lead dilatation time) was prolonged, complicated procedures (with technical problems) occurred slightly more often, and more advanced tools were required.

This is partly due to longer implant duration (VDD system: 129.9 months, VDD leads in other systems: 143.3 months, systems without VDD leads: 99.5 months), partly to the more frequent presence of abandoned leads (all systems containing VDD leads: 22.33% and all systems without VDD leads: 10.77%), and partly to the younger age of patients with VDD leads (51.87 vs. 57.74 years) at the time of system implantation. It results from a burden load of connective tissue on the leads in younger patients, which results in adhesions of the leads to right-sided heart structures [40,42]. VDD lead extraction does not increase the risk of major complications. A specific design of VDD leads combined with individual operator skills and team experience, as well as certain non-standard maneuvers, can facilitate the achievement of favorable results, even with older models of VDD leads.

Despite some disadvantages of VDD systems, including possible atrial undersensing in 4% to 16% [8,9,10,11] or the development of de novo sinus node disease [8] and the possible subsequent need for an upgrade to DDD systems, they are still used because of their shorter implantation time and lower complication rate [8,12].

However, upgrading procedures exposes the patient to a substantial risk of complications and a higher risk of device-related infection [45]. Marchandise et al. showed no difference in overall complication rate between DDD and VDD (6.1% vs. 9.1%) [12], but Schurrab et al. showed a lower overall rate of adverse events in the VDD group in comparison to the DDD group (9.6% vs. 11.6%) [13]. Wiegand et al. reported longer fluoroscopic time during DDD system implantation than during VDD system implantation, i.e., 9 min vs. 4.1 min [44], which was corroborated by other investigators [13]. Additionally, the costs of uncomplicated implantation of VDD systems were lower than the costs of DDD system implantation [13].

The leads should ensure sufficiently good detection of atrial potentials, a possibly long service life (resistance to mechanical damage and physicochemical factors), ease of extraction, and the possibility of placing their tips in the hemodynamically optimal place in the right ventricle. The disadvantages of VDD pacing leads include a fixed tip-atrial ring distance, which in practice eliminates the possibility of septal pacing. The passive mechanism of lead tip fixation (except in the latest models) also forces pacing from the apex of the right ventricle.

Currently, we cannot say how often such leads are removed and what the outcomes are (TLE procedure complexity and result). When reviewing the literature on TLE complications and lead extraction difficulties, we noticed that “VDD leads” are not even mentioned in the vast majority of papers. Based on three large studies, it can be assumed that VDD leads are removed in about 2–5% of all TLEs [32,33]. However, there is only one study proving the increased difficulty of VDD lead extraction [34]. Harunari et al. examined the factors affecting the outcome of lead extraction with an excimer laser sheath based on the extraction of 372 leads from 176 patients (11 VDD leads). The mean implant duration was 7.1 years. The procedural failure group had a longer time from implantation, a longer fluoroscopy time, and more VDD leads compared to the clinical success group. The investigators concluded that the presence of a VDD lead is an adverse factor for lead extraction, and when removing VDD leads, an operator should pay special attention to the procedure. The findings of our study in 103 patients undergoing extraction of VDD leads seem to confirm Harunari’s observations. Although the TLE procedure may be difficult and complicated, as is the case with leads with a long body dwell time and leads that are more difficult to extract due to their construction, discharge home on the same day is feasible and safe for selected patients (i.e., those without device infection and when the TLE procedure is completed in the morning) [45].

## 6. Conclusions

1. VDD leads are relatively rarely extracted during TLE procedures (3.42%). They have a longer implant duration (135.2 months) compared to all extracted leads in other systems without VDD leads (99.55 months), and abandoned leads are more common in patients with VDD leads (22.33% vs. 10.61%).
2. If VDD leads are removed, procedure duration (lead dilatation time) is longer, complicated procedures (so-called “technical problems”) occur slightly more often, and more advanced tools are required, but VDD lead extraction does not increase the risk of major complications.
3. Obtained data suggest that a specific design of VDD leads combined with individual operator skills and team experience, as well as certain non-standard maneuvers, can facilitate the achievement of favorable results, even with older models of VDD leads.

## 7. Study limitations

This study has some limitations. This is a retrospective analysis of prospectively (routinely) collected data. All procedures were performed using all types of mechanical systems, but not laser-powered sheaths. This study was aimed at assessing the effectiveness and outcome of VDD lead extraction. However, major complications concern the entire procedure and not the extraction of one type of lead(s). Therefore, we can never be sure which lead causes the complications during extraction. Patients with VDD leads often had abandoned leads or newer leads implanted during system upgrades. Also, difficulties during the procedure were caused not only by the mere removal of a given lead but also by the presence of additional leads. Finally, this is a presentation of a single, very experienced center. Therefore, the outcomes of the extraction procedures may not represent the overall safety and efficacy of transvenous extraction of leads, especially with a long implant duration.

## Figures and Tables

**Table 1:** Basic characteristics of pacing systems containing VDD leads for extraction and other CIEDs without VDD leads, with particular emphasis on the presence of abandoned leads.

Patients with VDD Leads	All Patients *N* (%)	Patients with Abandoned VDD Leads *N* (% of All)
Number of patients with VDD leads	103 (100.0)	23 (22.33)
Number of patients with active VDD systems	72 (69.90)	8 (11.11)
Other than VDD systems:	31 (30.10)	15 (48.39)
VVI system	3 (2.91)	3 (100.0)
DDD system	19 (18.45)	6 (31.58)
CRT-P system	3 (2.91)	0 (0.00)
ICD system	4 (3.88)	4 (100.0)
CRT-D system	2 (1.94)	2 (100.0)
Dwell time of active VDD leads being extracted [months]	129.9 ± 66.36	
Extraction time of VDD lead (other systems)	143.3 ± 48.89	
Extraction time of VDD lead (all)	135.2 ± 61.84	
Indications for TLE in 72 patients with VDD systems (without abandoned VDD leads)		
Systemic infection	13 (18.06)	
Local (pocket) infection	3 (4.18)	
Mechanical lead damage (electrical failure)	23 (31.94)	
Lead dysfunction (exit/entry block, dislodgement, extracardiac pacing, usually dry perforation)	17 (23.61)	
Change of pacing mode/upgrading, downgrading	9 (12.50)	
Other non-infectious indications *	7 (9.72)	
Indications for TLE in 31 patients with VDD abandoned leads		
Systemic infection	10 (32.26)	
Local (pocket) infection	1 (3.23)	
Mechanical lead damage (electrical failure)	10 (32.26)	
Lead dysfunction (exit/entry block, dislodgement, extracardiac pacing, usually dry perforation)	2 (6.45)	
Change of pacing mode/upgrading, downgrading	1 (3.23)	
Other non-infectious indications *	7 (22.59)	
Patients without VDD leads	All patients *N* (%)	Patients with abandoned leads *N* (%)
VVI system	470 (12.01)	119 (25.32)
AAI system	273 (7.17)	21 (7.69)
DDD or CRTP system	1866 (49.00)	162 (8.68)
Other (ICD, CRT-D)	1096 (28.78)	97 (8.85)
All patients without VDD leads	3705 (100.0)	399 (10.77)
Extraction time of all leads	99.55 ± 75.28	
ALL analyzed TLE procedures	3808 (100.0)	422 (11.08)

VDD—atrial sensing; ventricular sensing/pacing lead; CIED—cardiac implantable electronic device; VVI—single chamber pacemaker with ventricular sensing/pacing lead; DDD—dual chamber pacemaker; CRT-P—cardiac resynchronization therapy pacemaker; ICD—implantable cardioverter defibrillator; CRT-D—cardiac resynchronization cardioverter defibrillator; TLE—transvenous lead extraction; * Abandoned lead/prevention of abandonment (AF, redundant leads); threatening/potentially threatening lead (loops, free ends, left heart, LDTVD); other (MRI indications, cancer, painful pocket, loss of indications for pacing/ICD); and re-establishing venous access (symptomatic occlusion, SVC syndrome, lead replacement/upgrading); ±—standard deviation.

**Table 2:** Clinical patient data and indications for TLE in this study groups.

	Patients with VDD Pacing System or Presence of Abandoned VDD Lead	Patients with VVI Pacing System without VDD Lead	Patients with DDD or CRT-P Pacing Systems without VDD Lead	Patients with AAI Pacing Systems without VDD Lead	Patients with ICD-V, ICD-D, CRT-D Pacing Systems without VDD Lead
	*N* = 103 Mean ± SD *N* (%)	*N* = 470 Mean ± SD *N* (%)	*N* = 1866 Mean ± SD *N* (%)	*N* = 273 Mean ± SD *N* (%)	*N* = 1096 Mean ± SD *N* (%)
Patient age during TLE [years]	62.89 ± 16.57	63.68 ± 21.84 *p* = 0.079	67.97 ± 15.18 *p* = 0.002	71.01 ± 11.71 *p* = 0.001	62.81 ± 13.32 *p* = 0.241
Patient age at first system implantation [years]	51.74 ± 18.01	53.03 ± 23.64 *p* = 0.059	58.91 ± 17.2 *p* < 0.001	59.97 ± 13.09 *p* = 0. 001	57.16 ± 13.81 *p* = 0.018
Female	45 (43.70)	184 (39.15) *p* = 0.459	855 (45.86) *p* = 0.745	162 (59.31) *p* = 0.009	210 (19.18) *p* = 0.001
Ischaemic heart disease	52 (50.48)	227 (48.30) *p* = 0.769	1059 (56.78) *p* = 0.249	152 (55.68) *p* = 0.432	625 (57.08) *p* = 0.236
NYHA functional class III or IV	15 (15.46)	73 (15.53) *p* = 0.923	243 (14.03) *p* = 0.765	12 (4.30) *p* = 0.002	384 (35.07) *p* < 0.001
LVEF [%]	51.88 ± 13.27	52.86 ± 12.80 *p* = 0.822	53.91 ± 13.41 *p* = 0.193	56.98 ± 9.71 *p* = 0.002	38.35 ± 15.15 *p* < 0.001
Charlson co-morbidity index [points]	4.07 ± 3.84	4.39 ± 3.82 *p* = 0.247	4.67 ± 3.62 *p* = 0.017	4.45 ± 3.07 *p* = 0.033	5.12 ± 3.85 *p* = 0. 001
Main indications for TLE—(primary/predominant)					
Infective endocarditis with or without pocket infection	23 (22.33)	83 (17.66) *p* = 0.269	436 (23.38) *p* = 0.810	38 (13.92) *p* = 0.049	264 (24.02) *p* = 0.690
Local (isolated) pocket infection	4 (3.88)	58 (12.34) 0.012	178 (9.54) 0.054	18 (6.59) *p* = 0.318	100 (9.10) *p* = 0.071
Mechanical lead damage (electrical failure)	33 (32.04)	123 (26.17) *p* = 0.226	424 (22.73) *p* = 0.029	62 (22.71) *p* = 0.063	385 (35.13) *p* = 0.530
Lead dysfunction *	19 (18.45)	98 (20.85) *p* = 0.146	454 (24.45) *p* = 0.174	32 (11.72) *p* = 0.089	247 (22.54) *p* = 0.340
Change of pacing mode/upgrading, downgrading	10 (9.71)	42 (8.94) *p* = 0.860	140 (7.51) *p* = 0.411	25 (9.16) *p* = 0.869	24 (2.18) *p* < 0.001
Other non-infectious indication **	14 (13.59)	66 (14.04) *p* = 0.906	252 (13.51) *p* = 0.975	98 (35.90) *p* < 0.001	76 (6.92) *p* = 0.014

*p*—vs. patients with VDD lead; TLE—transvenous lead extraction; VDD—atrial sensing; ventricular sensing-pacing lead; VVI—single chamber pacemaker with ventricular sensing/pacing lead; DDD—dual chamber pacemaker; AAI—single chamber pacemaker with atrial sensing/pacing lead; ICD-V—implantable cardioverter defibrillator with ventricular defibrillation lead; ICD-D—dual chamber cardioverter defibrillator; CRT-P—cardiac resynchronization therapy pacemaker; CRT-D—cardiac resynchronization therapy cardioverter defibrillator; LVEF—left ventricular ejection fraction; * Lead dysfunction—exit/entry block; dislodgement; perforation; extracardiac pacing. ** Abandoned lead—prevention of abandonment (AF, redundant leads); threatening/potentially threatening lead (loops, free ending, left heart, LDTVD), other (MRI indications, cancer, painful pocket, loss of indications for pacing/ICD); and re-establishing venous access (symptomatic occlusion, SVC syndrome, lead replacement/upgrading); ± SD—standard deviation.

**Table 3:** Comparison of CIED-related risk factors for TLE difficulty, major complications, and scores for prediction of increased procedure complexity (in 3808 patients).

	Patients with VDD Pacing System or Presence of Abandoned VDD Lead	Patients with VVI Pacing System without VDD Lead	Patients with DDD or CRTP Pacing Systems without VDD Lead	Patients with AAI Pacing System without VDD Lead	Patients with ICD-V, ICD-D, CRT-D Pacing Systems without VDD Lead
	*N* = 103 Mean ± SD *N* (%)	*N* = 470 Mean ± SD *N* (%)	*N* = 1866 Mean ± SD *N* (%)	*N* = 273 Mean ± SD *N* (%)	*N* = 1096 Mean ± SD *N* (%)
System-related risk factors for major complications or increased procedure complexity					
Longest lead dwell time before TLE [months]	135.2 ± 61.84	127.3 ± 75.07 *p* = 0.027	109.3 ± 79.43 *p* < 0.001	133.20 ± 75.96 *p* = 0.509	68.64 ± 48.24 *p* < 0.001
Global lead dwell time before TLE [years]	14.55 ± 9.14	13.52 ± 12.35 *p* = 0.004	18.77 ± 14.38 *p* = 0.016	16.61 ± 11.53 *p* = 0.243	10.30 ± 8.97 *p* < 0.001
Presence of abandoned leads before TLE	23 (22.33)	119 (25.32) *p* = 0.610	162 (8.69) *p* < 0.001	21 (7.69) *p* = 0.002	97 (8.85) *p* < 0.001
Number of leads in the heart before TLE	1.58 ± 0.86	1.32 ± 0.61 *p* = 0.025	2.22 ± 0.52 *p* < 0.001	1.53 ± 0.64 *p* = 0.542	1.92 ± 0.87 *p* < 0.001
≥4 leads in the heart before TLE	3 (2.91)	3 (0.64) *p* = 0.129	69 (3.70) *p* = 0.885	2 (0.73) *p* = 0.254	41 (3.74) *p* = 0.877
Leads on both sides of the chest before TLE	8 (7.77)	27 (5.75) *p* = 0.569	51 (2.74) *p* < 0.009	3 (1.10) *p* = 0.002	18 (1.64) *p* = 0.804
Number of procedures before lead extraction	2.17 ± 1.01	1.98 ± 1.12 *p* = 0.057	1.85 ± 1.09 *p* < 0.001	2.03 ± 1.10 *p* = 0.164	1.71 ± 0.95 *p* < 0.001
Various scores predicting the risk of major complications or procedure complexity					
SAFeTY-TLE score estimated risk of MC [%]	1.67 ± 2.70	1.81 ± 3.05 *p* = 0. 779	2.12 ± 3.44 *p* = 0.264	2.11 ± 2.72 *p* = 0.067	0.87 ± 1.56 *p* < 0.001
Average EROS score [points]	1.74 ± 0.84	1.74 ± 0.83 *p* = 0.826	1.58 ± 0.77 *p* = 0. 148	1.59 ± 0.83 *p* = 0.152	1.33 ± 0.51 *p* < 0.001
MB score [points]	2.79 ± 0.99	2.27 ± 1.20 *p* < 0.001	2.59 ± 1.27 *p* = 0.959	2.64 ± 1.11 *p* = 0.429	2.68 ± 1.26 *p* = 0.301
LED index [points]	12.54 ± 5.31	11.53 ± 7.47 *p* < 0.001	10.65 ± 6.75 *p* < 0.001	12.26 ± 6.39 *p* = 0.380	7.60 ± 4.32 *p* < 0.001
Advanced TLE (Mazzone) scale, average values [points]	1.94 ± 0.71	1.55 ± 0.77 *p* < 0.001	2.01 ± 0.82 *p* = 0.234	1.70 ± 0.76 *p* = 0.012	2.75 ± 0.87 *p* < 0.001
LECOM score [points]	10.06 ± 3.57	8.49 ± 4.43 *p* < 0.001	8.31 ± 4.19 *p* < 0.001	8.02 ± 3.86 *p* < 0.001	6.81 ± 3.69 *p* < 0.001
LECOM score [%]	27.61 ± 17.64	22.94 ± 20.02 *p* < 0.001	22.87 ± 19.68 *p* < 0.001	20.71 ± 17.21 *p* < 0.001	16.15 ± 16.26 *p* < 0.001
TLE procedure-related risk factors for major complications and procedure complexity					
Number of extracted leads per patient	1.45 ± 0.75	1.23 ± 0.53 *p* = 0.044	1.86 ± 0.68 *p* < 0.001	1.42 ± 0.55 *p* = 0.430	1.56 ± 0.80 *p* = 0.156
Extraction of abandoned lead(s) (any)	22 (21.36)	114 (24.26) *p* = 0.619	148 (7.94) *p* < 0.001	17 (6.22) *p* < 0.001	81 (7.40) *p* < 0.001
Extraction of passive fixation leads (excluding LV leads)	103 (100.0)	339 (72.13) *p* < 0.001	1141 (61.18) *p* < 0.001	162 (59.34) *p* < 0.001	472 (43.11) *p* < 0.001
Oldest extracted lead per patient [months]	135.2 ± 61.84	124.7 ± 88.27 *p* = 0.013	107.3 ± 78.23 *p* < 0.001	130.8 ± 75.48 *p* = 0.316	67.80 ± 48.24 *p* < 0.001
Average extracted lead dwell time per patient [months]	122.2 ± 57.17	119.2 ± 82.56 *p* = 0.097	102.2 ± 69.67 *p* < 0.001	127.2 ± 71.04 *p* = 0.931	64.32 ± 43.56 *p* < 0.001

*p*—vs. patients with VDD lead; TLE—transvenous lead extraction; VDD—atrial sensing; ventricular sensing-pacing lead; VVI—single chamber pacemaker with ventricular sensing/pacing lead; DDD—dual chamber pacemaker; AAI—single chamber pacemaker with atrial sensing/pacing lead; ICD-V—implantable cardioverter defibrillator with ventricular defibrillation lead; ICD-D—dual chamber cardioverter defibrillator; CRTP—cardiac resynchronization therapy pacemaker; CRT-D—cardiac resynchronization therapy cardioverter defibrillator; SAFeTY-TLE calculator of risk of major complications (MC)—risk of MC in%; MC—major complications; EROS score—increased risk of significant procedural complications that require urgent surgical intervention (1–3); LECOM score—combined: lead dilatation time, use of second line or advanced tools, and advanced techniques; MB score—the need for advanced tools to achieve TLE success; LED index—difficult TLE defined by fluoroscopy time; LV—left ventricle; ± SD—standard deviation.

**Table 4:** Procedure complexity in this study groups.

	Patients with VDD Pacing System or Presence of Abandoned VDD Lead	Patients with VVI Pacing System without VDD Lead	Patients with DDD or CRTP Pacing Systems without VDD Lead	Patients with AAI Pacing System without VDD Lead	Patients with ICD-V, ICD-D, CRT-D Pacing Systems without VDD Lead
	*N* = 103 Mean ± SD *N* (%)	*N* = 470 Mean ± SD *N* (%)	*N* = 1866 Mean ± SD *N* (%)	*N* = 273 Mean ± SD *N* (%)	*N* = 1096 Mean ± SD *N* (%)
TLE complexity and outcomes					
Procedure duration (sheath-to-sheath) [minutes]	19.81 ± 23.73	14.70 ± 20.56 *p* = 0.039	16.48 ± 24.39 *p* < 0.001	11.46 ± 12.70 *p* = 0.038	12.97 ± 22.13 *p* = 0.001
* Average time of single lead extraction [minutes]	14.55 ± 29.52	11.30 ± 13.20 *p* = 0.286	8.52 ± 11.43 *p* < 0.001	8.54 ± 10.11 *p* = 0.003	8.06 ± 11.96 *p* = 0.001
Number of patients with any technical problem	29 (28.15)	136 (28.93) *p* = 0.969	597 (32.07) *p* = 0.491	55 (56.85) *p* = 0.001	212 (19.36) *p* = 0.046
Number of technical problems per patient	0.30 ± 0.70	0.29 ± 0.60 *p* = 0.785	0.32 ± 0.71 *p* = 0.678	0.20 ± 0.57 *p* = 0.501	0.19 ± 0.55 *p* = 0.403
Two or more technical problems	10 (9.71)	29 (6.17) *p* = 0.282	144 (7.72) *p* = 0.587	10 (3.66) *p* = 0.038	39 (3.56) *p* = 0.006
Use of additional tools, Evolution (old and new), or TightRail	5 (4.85)	12 (2.55) *p* = 0.354	27 (1.45) *p* = 0.024	1 (0.37) *p* = 0.008	13 (1.19) *p* = 0.012
Metal sheaths	4 (3.88)	47 (10.00) *p* = 0.075	167 (8.95) *p* = 0.110	22 (8.06) *p* = 0.232	70 (6.40) *p* = 0.425
Lasso catheters/snares, basket catheters	10 (9.71)	43 (9.15) *p* = 0.992	112 (6.02) *p* = 0.191	8 (2.93) *p* = 0.013	20 (1.83) *p* = 0.001
Need to change the venous approach	5 (4.85)	26 (5.53) *p* = 0.972	75 (4.02) *p* = 0.873	7 (2.56) *p* = 0.432	13 (1.19) *p* = 0.012
CID-TLE score (dilatation time, use of second-line tools, advanced tools, and advanced techniques) [points 0–5]	0.83 ± 1.32	0.76 ± 1.28 *p* = 0.403	0.60 ± 1.17 *p* = 0.208	0.40 ± 1.00 *p* = 0.028.	0.39 ± 0.92 *p* = 0.016
CID-TLE score—the combined difficulty score: 2 and more points	10 (9.71)	66 (14.04) *p* = 0.311	206 (11.05) *p* = 0.794	20 (7.33) *p* = 0.584	54 (4.93) *p* = 0.067

*p*—vs. patients with VDD lead; TLE—transvenous lead extraction; VDD—atrial sensing; ventricular sensing-pacing lead; VVI—single chamber pacemaker with ventricular sensing/pacing lead; DDD—dual chamber pacemaker; AAI—single chamber pacemaker with atrial sensing/pacing lead; ICD-V—implantable cardioverter defibrillator with ventricular defibrillation lead; ICD-D—dual chamber cardioverter defibrillator; CRTP—cardiac resynchronization therapy pacemaker; CRT-D—cardiac resynchronization therapy cardioverter defibrillator; * average time of single lead extraction—sheath-to-sheath time/number of extracted leads; the Complex Indicator of the Difficulty of the TLE (CID-TLE); ± SD—standard deviation.

**Table 5:** Procedure complications and long-term outcomes in patients with and without VDD leads to extraction.

	Patients with VDD Pacing System or Presence of Abandoned VDD Lead	Patients with VVI Pacing without VDD Lead	Patients with DDD or CRTP Pacing Systems without VDD Lead	Patients with AAI Pacing System without VDD Lead	Patients with ICD-V, ICD-D, CRT-D Pacing Systems without VDD Lead
	*N* = 103 Mean ± SD *N* (%)	*N* = 470 Mean ± SD *N* (%)	*N* = 1866 Mean ± SD *N* (%)	*N* = 273 Mean ± SD *N* (%)	*N* = 1096 Mean ± SD *N* (%)
TLE efficacy and complications					
Major complications (any)	2 (1.94)	11 (2.34) *p* = 0.905	50 (2.68) *p* = 0.889	7 (2.56) *p* = 0.979	7 (0.64) *p* = 0.390
Haemopericardium	0 (0.00)	7 (1.49) *p* = 0.453	34 (1.82) *p* = 0.320	4 (1.47) *p* = 0.502	4 (0.37) *p* = 0.780
Haemothorax	1 (0.97)	0 (0.00) *p* = 0.404	0 (0.00) *p* = 0.044	2 (0.73) *p* = 0.676	2 (0.18) *p* = 0.618
Tricuspid valve damage during TLE (severe)	0 (0.00)	5 (1.06) *p* = 0.691	15 (0.89) *p* = 0.740	1 (0.37) *p* = 0.612	1 (0.09) *p* = 0.140
Rescue cardiac surgery	1 (0.97)	6 (1.12) *p* = 0.811	28 (1.50) *p* = 0.988	5 (1.83) *p* = 0.895	5 (0.46) *p* = 0.982
Death, procedure related (intra-, post-procedural)	0 (0.00)	3 (0.64) *p* = 0.953	1 (0.05) *p* = 0.814	0 (0.00) N	9 (0.82) *p* = 0.744
Partial radiograpic success (retained tip or <4 cm lead fragment)	6 (5.83)	29 (6.17) *p* = 0.926	100 (5.36) *p* = 0.983	8 (2.93) *p* = 0.309	18 (1.64) *p* = 0.012
Procedural success	96 (93.20)	435 (92.55) *p* = 0.983	1752 (93.94) *p* = 0.926	264 (96.70) *p* = 0.107	1076 (98.27) *p* = 0.003
Survival after the TLE procedure during 2092 ± 1462 [1–6239] days of follow-up					
Survivors	64 (62.14)	256 (54.47) Log rank *p* < 0.001	1207 (64.72) Log rank *p* = 0.118	176 (64.47) Log rank *p* = 0.339	591 (53.97) Log rank *p* < 0.001
All deaths	39 (37.86)	214 (45.53) Log rank *p* < 0.001	658 (35.28) Log rank *p* = 0.118	97 (35.53) Log rank *p* = 0.339	504 (46.03) Log rank *p* < 0.001

*p*—vs. patients with VDD lead; TLE—transvenous lead extraction; VDD—atrial sensing; ventricular sensing-pacing lead; VVI—single chamber pacemaker with ventricular sensing/pacing lead; DDD—dual chamber pacemaker; AAI—single chamber pacemaker with atrial sensing/pacing lead; ICD-V—implantable cardioverter defibrillator with ventricular defibrillation lead; ICD-D—dual chamber cardioverter defibrillator; CRTP—cardiac resynchronization therapy pacemaker; CRT-D—cardiac resynchronization therapy cardioverter defibrillator; N—non-comparable; ± SD—standard deviation.

**Table 6:** Predictors of lead extraction complexity and clinical success.

	Univariable Regression			Multivariable Regression		
	OR	95%CI	*p*	OR	95%CI	*p*
High procedure complexity						
Patient age at first system implantation [by 1 year]	0.966	0.962–0.971	<0.001	0.978	0.972–0.983	<0.001
Number of leads in the heart [by 1]	2.076	1.860–2.316	<0.001	1.726	1.448–2.059	<0.001
Passive fixation leads [y/n]	3.640	2.989–4.434	<0.001	1.535	1.219–1.933	<0.001
Abandoned lead(s) [y/n]	6.561	5.289–8.140	<0.001	2.015	1.449–2.801	<0.001
Dwell time of the oldest lead [by 1 year]	1.154	1.139–1.170	<0.001	1.109	1.091–1.128	<0.001
VDD pacing system or presence of abandoned VDD leads [y/n]	1.859	1.212–2.854	0.005	1.599	0.766–3.337	0.211
VVI pacing system [y/n]	1.464	1.166–1.840	0.001	1.370	0.717–2.620	0.340
AAI pacing system [y/n]	0.570	0.393–0.829	0.003	0.680	0.341–1.357	0.274
DDD pacing system [y/n]	1.255	1.067–1.476	0.006	1.149	0.672–1.964	0.612
CRTP pacing system [y/n]	0.885	0.529–1.482	0.642			
ICD/CRTD system [y/n]	0.638	0.526–0.774	<0.001	1.334	0.775–2.294	0.298
Clinical success						
Patient age at first system implantation [by 1 year]	1.027	1.016–1.038	<0.001	1.017	1.003–1.030	0.014
Number of leads in the heart [by 1]	0.510	0.398–0.654	<0.001	0.615	0.430–0.879	0.008
Lead passive fixation [y/n]	0.247	0.133–0.459	<0.001	0.524	0.268–1.023	0.058
Abandoned lead(s) presence [y/n]	0.231	0.142–0.374	<0.001	0.761	0.369–1.568	0.459
Dwell time of the oldest lead [by 1 year]	0.905	0.882–0.929	<0.001	0.954	0.921–0.987	0.007
VDD pacing system or presence of abandoned VDD leads [y/n]	2.185	0.301–15.88	0.440			
VVI pacing system presence [y/n]	0.636	0.354–1.144	0.131			
AAI pacing system presence [y/n]	1.437	0.522–3.955	0.483			
DDD pacing system presence [y/n]	0.561	0.355–0.887	0.013	1.016	0.593–1.742	0.954
CRTP pacing system presence [y/n]	0.515	0.185–1.435	0.204			
ICD/CRTD system presence [y/n]	3.599	1.726–7.506	<0.001	2.464	1.037–5.855	0.041
Major complications						
Patient age at first system implantation [by 1 year]	0.969	0.959–0.979	<0.001	0.986	0.973–1.000	0.048
Number of leads in the heart [by 1]	1.657	1.276–2.151	<0.001	1.188	0.769–1.834	0.437
Passive fixation leads [y/n]	3.351	1.872–5.998	<0.001	1.189	0.620–2.279	0.603
Abandoned lead(s) [y/n]	3.829	2.341–6.265	<0.001	1.388	0.624–3.089	0.421
Dwell time of the oldest lead [by 1 year]	1.144	1.116–1.173	<0.001	1.109	1.072–1.146	0.000
VDD pacing system or presence of abandoned VDD leads [y/n]	0.936	0.233–3.754	0.926			
VVI pacing system [y/n]	1.170	0.616–2.225	0.631			
AAI pacing system [y/n]	1.284	0.585–2.819	0.532			
DDD pacing system [y/n]	2.232	1.394–3.576	<0.001	1.525	0.796–2.921	0.203
CRTP pacing system [y/n]	0.001	0.000–0.002	0.980			
ICD/CRTD system [y/n]	0.239	0.110–0.522	<0.001	0.653	0.254–1.684	0.378

VDD—atrial sensing; ventricular sensing-pacing lead; VVI—single chamber pacemaker with ventricular sensing/pacing lead; DDD—dual chamber pacemaker; AAI—single chamber pacemaker with atrial sensing/pacing lead; ICD—implantable cardioverter defibrillator; CRTP—cardiac resynchronization therapy pacemaker; CRTD—cardiac resynchronization therapy cardioverter defibrillator; ± SD—standard deviation.

## Data Availability

Data are available upon request to authors.
